# MiR-122 Targets SerpinB3 and Is Involved in Sorafenib Resistance in Hepatocellular Carcinoma

**DOI:** 10.3390/jcm8020171

**Published:** 2019-02-01

**Authors:** Cristian Turato, Francesca Fornari, Daniela Pollutri, Matteo Fassan, Santina Quarta, Gianmarco Villano, Mariagrazia Ruvoletto, Luigi Bolondi, Laura Gramantieri, Patrizia Pontisso

**Affiliations:** 1 Venetian Institute of Oncology (IOV-IRCCS), 35128 Padua, Italy; cristianturato@gmail.com; 2 Center for Applied Biomedical Research, St. Orsola-Malpighi University Hospital, 40138 Bologna, Italy; francesca.fornari2@unibo.it (F.F.); danielapollutri@hotmail.it (D.P.); luigi.bolondi@unibo.it (L.B.); laura.gramantieri@aosp.bo.it (L.G.); 3 Department of Medicine, University of Padua, 35128 Padua, Italy; matteo.fassan@unipd.it (M.F.); santina.quarta@unipd.it (S.Q.); mariagrazia.ruvoletto@unipd.it (M.R.); 4 Department of Surgical, Gastroenterological and Oncological Sciences, University of Padua, 35128 Padua, Italy; gianmarco.villano@unipd.it

**Keywords:** hepatocellular carcinoma, micro RNA, molecular targets

## Abstract

The only first-line treatment approved for advanced hepatocellular carcinoma (HCC) is sorafenib. Since many patients experience drug resistance, the discovery of more effective therapeutic strategies represents an unmet clinical need. MicroRNA (MiR)-122 is downregulated in most HCCs, while oncogenic SerpinB3 is upregulated. Here, we assessed the relationship between miR-122 and SerpinB3 and their influence on cell phenotype and sorafenib resistance in HCC. A bioinformatics analysis identified SerpinB3 among hypothetical miR-122 targets. In SerpinB3-overexpressing HepG2 cells, miR-122 transfection decreased SerpinB3 mRNA and protein levels, whereas miR-122 inhibition increased SerpinB3 expression. Luciferase assay demonstrated the interaction between miR-122 and SerpinB3 mRNA. In an HCC rat model, high miR-122 levels were associated with negative SerpinB3 expression, while low miR-122 levels correlated with SerpinB3 positivity. A negative correlation between miR-122 and SerpinB3 or stem cell markers was found in HCC patients. Anti-miR-122 transfection increased cell viability in sorafenib-treated Huh-7 cells, while miR-122 overexpression increased sorafenib sensitivity in treated cells, but not in those overexpressing SerpinB3. In conclusion, we demonstrated that miR-122 targets SerpinB3, and its low levels are associated with SerpinB3 positivity and a stem-like phenotype in HCC. MiR-122 replacement therapy in combination with sorafenib deserves attention as a possible therapeutic strategy in SerpinB3-negative HCCs.

## 1. Introduction

Hepatocellular carcinoma (HCC), the major form of primary liver tumors, is one of the most common cancers worldwide. The incidence and mortality of this liver malignancy is increasing in most areas of the world as a consequence of aging and emerging new risk factors such as metabolic syndrome, as well as the recognized role of factors including viral infections, obesity, and alcohol abuse [1,2]. Although new advances in genomics provide an increasingly comprehensive understanding of HCC development, the molecular pathogenesis of HCC remains poorly understood, and the clinical heterogeneity of HCC together with the lack of diagnostic biomarkers and treatment strategies contribute to the high mortality rate recorded for this aggressive tumor [3]. For these reasons, research and development for new effective targeted therapies still represent a major clinical need. MicroRNAs (miRNAs) are a class of single-stranded non-coding RNAs of 19–25 nucleotides that serve as negative regulators of gene expression by interacting with 3’untranslated regions (3’UTRs) of the target genes [4]. They are involved in various biological and pathological processes and their deregulation has been often associated with disease progression and cancer [5,6].

In the liver, miR-122 is the most abundant miRNA, constituting 70% of the total hepatic miRNAs [7,8]. miR-122 is crucial not only determining the normal liver function but it also plays pivotal roles in various liver diseases, including viral hepatitis C, where it has been involved in Hepatitis C Virus (HCV) replication [9]. Moreover, miR-122 has been reported to be down-regulated in preneoplasic nodules and HCCs and inversely associated with metastasis formation and poor prognosis [10,11]. In fact, mice with germline or liver-specific knockout of miR-122 inevitably develop tumors resembling HCC [12]. Although different genes have been proposed as targets of miR-122, the mechanism behind miR-122 regulation of tumorigenesis in HCCs remains poorly understood [13,14,15]. Recently, we reported the expression profile of the best-characterized miRNAs in liver cancer cells and we identified some tumor-suppressive miRNAs, including miR-122, which are modulated by the ov-serpin SerpinB3 [16]. This molecule, which is not detectable in normal hepatocytes, has been found progressively upregulated in liver cirrhosis, dysplastic nodules, and hepatocellular carcinoma [17], especially in those with early recurrence and poor prognosis [18]. Concerning its biological effects, it has been found to induce apoptosis resistance, increased cell proliferation, and cell invasiveness [19]; however its relationship with HCC-specific miRNAs is not yet clear.

In the present study, we have explored the possible relationship between miR-122 and SerpinB3 by using in vivo and in vitro approaches. To this aim, we have used a diethyl nitrosamine (DEN)-induced HCC rat model and the results have been compared to those found in human HCC specimens. In addition, a subset of HCC cell lines overexpressing SerpinB3 or transfected with miR-122 were analyzed, in order to better understand the interplay between these two molecules in liver tumors as well as their involvement in sorafenib resistance.

## 2. Materials and Methods

### 2.1. Patients

Two independent groups of patients were used in this study. A first group (*n* = 35) from St. Orsola-Malpighi University Hospital was used for gene expression analysis and a second group (*n* = 42) from University of Padua was used in tissue microarray analysis. Firstly, HCC and cirrhotic tissues were obtained from 35 randomly selected patients (30 males and 5 females, median age 69 years, range 51–81 years) undergoing liver resection for HCC. Tissues were collected at surgery and were stored as previously described [20]. Secondly, 42 HCCs and their matched cirrhotic tissue (35 males and 7 females, median age 65.8 years, range 46.8–86.4 years) were processed using the Galileo CK3500 Arrayer (Integrated Systems Engineering, Milan, Italy), a semiautomatic and a computer-assisted tissue microarray (TMA) platform. Two tissue cores (1 mm in diameter) were obtained from each considered lesion.

Local ethics committees approved the studies and all patients signed an informed consent. Histopathologic grading was scored according to Edmondson and Steiner criteria. No patient received anticancer treatment prior to surgery. The research was conducted ethically in accordance with the World Medical Association Declaration of Helsinki. Subjects gave their written informed consent. The research institute’s committee on human research approved the study protocol. Animal experiments conform to internationally accepted standards and have been approved by the appropriate institutional review body.

### 2.2. Cell Lines

HepG2, Hep3B (ATCC, LGC Standards S.r.l., Milan, Italy), and Huh7 cell lines (kindly provided by Professor G Giannelli, University of Bari, Italy), derived from human hepatoma cells, were cultured as previously described [21]. HepG2 and Huh-7 cells were stably transfected with a plasmid vector carrying the wild-type SerpinB3 human gene as previously reported [19]. HCC-derived cell lines were transfected with 100 nmol/L of pre-miR-122-5p, anti-miR-122-5p, or negative control precursor and inhibitor miRNAs (Ambion, Austin, TX, USA) for 24 and 48 h. Oligonucleotide transfection was performed by using Lipofectamine 2000 (Life Technologies, Carlsbad, CA, USA) according to the manufacturer’s instructions. In addition, cell viability and the enzymatic activation of effector caspases 3 were evaluated in transfected HCC cells following multi-kinase inhibitor sorafenib administration (5 μM for 48 h) by using CellTiter-Glo and Caspase-Glo 3/7 assays (Promega, Madison, WI, USA) according to manufacturers’ instructions. These experiments assays were run in triplicate.

### 2.3. Luciferase Assay

A portion of the 3’UTR region of human SerpinB3 gene (586 bp) was amplified by PCR using primers and conditions reported in Supplementary Table S1 and cloned downstream of the reporter gene into the XbaI site. Luciferase reporter assay was performed in HepG2 cells as previously reported [22].

### 2.4. DEN-HCC Rat Model

The diethylnitrosamine (DEN)-induced HCC rat model was established as previously described [20]. RNA samples were extracted from frozen tissues of 17 DEN-HCC rats. Tissues were collected at sacrifice and were stored as previously described [20]. All animals received human care in accordance with the criteria published by the National Institutes of Health. The local ethics committee approved the research protocol (14/70/12).

### 2.5. Real-Time PCR

Total RNA was isolated from transfected HCC cells and from rat and human HCC specimens as previously described [10]. Quantification of miR-122-5p (ID: 002245) was obtained by using TaqMan miRNA assay (Applied Biosystems, Foster City, CA, USA). RNU6B (ID: 001093) was used as housekeeping gene for human samples, whereas 4.5S RNA(H) (ID: 001717) was used for samples of rat origin.

In addition SerpinB3, CD133 and EpCam mRNAs were quantified by quantitative real-time qPCR and were carried out as previously described using the CFX96 Real-Time instrument (Bio-Rad Laboratories Inc, Hercules, CA, USA) [23]. Relative gene expression was normalized to the housekeeping genes and was calculated using the 2^−ΔΔCT^ method. Primers and amplification conditions are reported in Supplementary Table S1.

### 2.6. Western Blot

Transfected HCC derived cell lines were lysed using the RIPA Lysis and Extraction Buffer (Life Technologies, Grand Island, NY, USA) supplemented with protease inhibitors (Roche, Indianapolis, IN). The total protein was quantified with a Pierce BCA Protein Assay Kit (Pierce Biotechnology, Rockford, IL, USA) according to the manufacturer’s protocol. Protein samples were separated by sodium dodecyl-sulfate-polyacrylamide gel electrophoresis (SDS-PAGE) and were transferred to a polyvinylidene fluoride (PVDF) membrane. After blocking in phosphate-buffered saline/Tween-20 containing 5% non-fat dry milk at room temperature for 1 h, the membrane was incubated at 4 °C overnight with primary antibodies: anti-SerpinB3 polyclonal antibody (1:500 Xeptagen SpA, Marghera, Venice, Italy), anti-PROM1/CD133 (1:1000, Cell Signaling Technology), anti-EpCAM (1:1000, Cell Signaling Technology), anti-cleaved caspase-3 (1:1000, Cell Signaling Technology), and anti-PARP (1:1000, Cell Signaling Technology). Then, the membrane was incubated with peroxidase-conjugated anti-rabbit immunoglobulins in Tris-buffered saline-Tween containing 5% non-fat dry milk.

Sample loading was evaluated by reblotting the same membrane with β-actin (1:1000, Sigma Aldrich) for total extract normalization. Proteins were visualized with LumiGLO chemiluminescent substrate (Cell Signaling Technology, Danvers, MA, USA) and chemiluminescent signal was detected by using ChemiDoc (Bio-Rad Laboratories Inc, Hercules, CA, USA). WB analysis was performed in triplicate.

### 2.7. MiR-122 In Situ Hybridization (ISH) and Immunohistochemical (IHC) Analysis

Locked nucleic acid (LNA) probes with complementarity to miR-122 were labeled with 5′-biotin and synthesized by Exiqon (Vedbaek, Denmark). Tissue microarray (TMA) sections were digested with ISH protease 1 (Ventana Medical Systems, Milan, Italy) and ISH was performed as previously described, with minor modifications [24]. Positive (U6; Exiqon) and negative scrambled LNA probes were used as controls. Only cytoplasmic miR-122 intensity was retained for scoring purposes. Protein/microRNA co-expression analysis was carried out as previously described with minor modifications [25]. After ISH staining, we used the Benchmark LT automated system from Leica Microsystems Bondmax (Leica, Wetzlar, Germany) according to the manufacturer’s specifications to perform the immunohistochemistry for SerpinB3 [21]. The expression of SerpinB3 was cytoplasmic. These experiments assays were run in duplicate and color-relative staining intensities were evaluated by ImageJ software evaluation.

### 2.8. Flow Cytometry

Immunophenotype of PROM1/CD133 and EpCAM (CD326) was performed by flow cytometry (Cytoflex S, Beckman Coulter, Brea, CA, USA) using CD133 monoclonal antibody (13A4)-APC and CD326 monoclonal antibody (MH99)-Alexa Fluor 488, respectively (eBioscience).

### 2.9. Statistical Analysis

Differences between groups were analyzed using Mann-Whitney test. Spearman rank correlation coefficient was used to explore associations between two variables. Reported *p*-values were two-sided and considered significant when lower than 0.05. Statistical calculations were carried out with Graph Pad InStat Software (San Diego, CA, USA). * *p* < 0.05, ** *p* < 0.01, *** *p* < 0.001.

## 3. Results

### 3.1. SerpinB3 Is a Target of miR-122 in HCC

**Figure 1:**
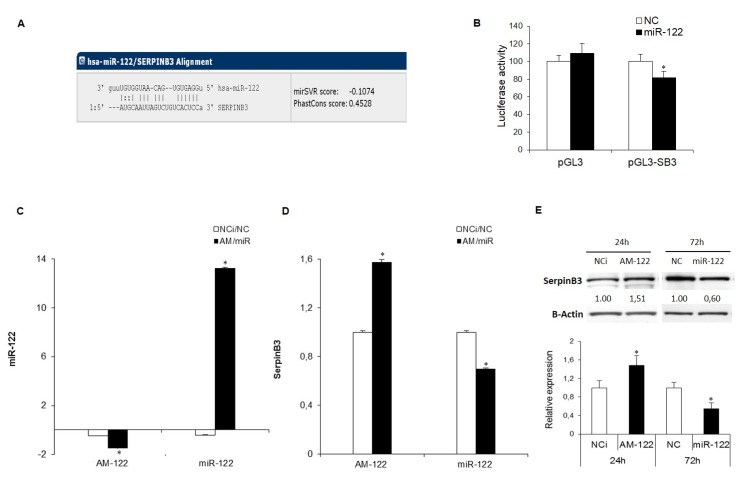
(**A**) miR-122 binding site in the SerpinB3 3’untranslated region (3′UTR) as reported by the TargetScan algorithm. (**B**) Dual-luciferase assay in HepG2 cells. The SerpinB3 3’UTR-containing vector was co-transfected with miR-122 or negative control (NC). MiR-122 overexpression determined a decrease of the reporter gene activity in pGL3-SerpinB3 co-transfected HepG2 cells. (**C**) qPCR analysis of miR-122 levels following miR-122 inhibitor (AM-122) or miR-122 mimic transfection in HepG2 cells stably overexpressing SerpinB3 (HepG2/SerpinB3) with respect to controls. U6RNA was used as housekeeping gene. Y-axis reports 2^−ΔΔCt^ levels expressed in logarithmic form. (**D**,**E**) qPCR and Western blot analysis of SerpinB3 in transfected HepG2/SerpinB3 cells. β-Actin was used as housekeeping gene. Y-axis reports 2^−ΔΔCt^ levels. NCi: miRNA inhibitor negative control; NC: miRNA precursor negative control. (* *p* ˂ 0.05). Data shown are representative results of at least three independent experiments.

In order to explore a possible relationship between miR-122 and SerpinB3, we performed a computational analysis that identified SerpinB3 as a miR-122 hypothetical target gene (TargetScan algorithm Figure 1A).

Moreover, to investigate the interaction between miR-122 and SerpinB3, the 3’UTR region of SerpinB3 mRNA was cloned downstream of a reporter gene and the resulting vector (pGL3-SerpinB3) was employed in a dual-luciferase assay. Co-transfection of pGL3-SerpinB3 vector with miR-122 in HepG2 cells determined a decrease of the reporter luciferase activity with respect to negative control transfected cells (*p* = 0.013) (Figure 1B). This cell line was chosen because of its low/intermediate miR-122 basal levels with respect to other HCC cell lines (Figure S1).

To evaluate further the regulation of SerpinB3 by miR-122, we performed a functional analysis by transfecting miR-122 or anti-miR-122 in SerpinB3-overexpressing HepG2 cells (Figure 1C). MiR-122 overexpression determined a significant decrease of SerpinB3 mRNA and protein levels with respect to control cells (Figure 1D,E). As expected, an opposite result was obtained in miR-122-silenced HepG2 cells, showing an upregulation of SerpinB3 at both mRNA and protein levels (Figure 1D,E). We confirmed these data in a second HCC cell line and we choose Huh-7 cells harboring high miR-122 levels (Figure S1A), genetically manipulated to stably overexpress SerpinB3. After transfection with miR-122 mimics a decrease of SerpinB3 mRNA levels was observed, whereas transfection with anti-miR-122 oligonucleotides determined an increase of SerpinB3 levels (Figure S1B,C). Taken together, these data demonstrate that miR-122 regulates SerpinB3 expression by causing its mRNA degradation.

### 3.2. MiR-122 and SerpinB3 Inversely Correlate In Vivo

**Figure 2:**
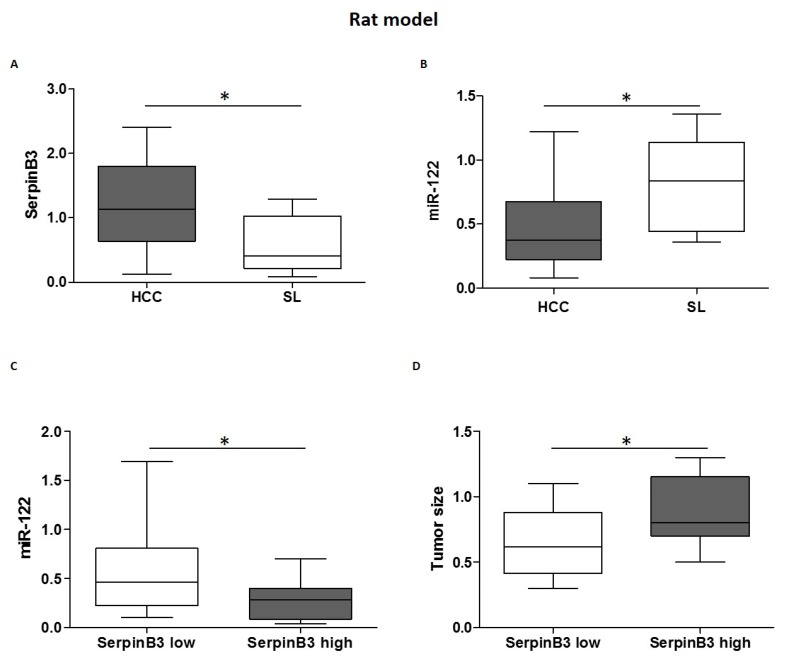
(**A**) SerpinB3 and (**B**) miR-122 expression in hepatocellular carcinoma (HCC) nodules and matched surrounding liver (SL) tissues from diethyl nitrosamine (DEN)-induced HCC rats. Y-axis reports 2^−ΔΔCt^ levels from qPCR analysis. The values are represents as box-and-whiskers graph with the minimum, maximum, and median data. (* *p* ˂ 0.05 Mann–Whitney test). (**C**) MicroRNA (MiR)-122 expression in tumor tissues from HCC rats in relation to SerpinB3 expression. HCCs were grouped on the basis of SerpinB3 expression as detected by qPCR analysis (* *p* ˂ 0.05 Mann–Whitney test). Specifically, the high SerpinB3 group included HCC specimens with a cycle threshold (Ct) value lower than 35, whereas the low SerpinB3 group included HCC specimens with a Ct value higher than 35. (**D**) Box plot graph representing tumor size in HCC rats in relation to SerpinB3 expression (* *p* ˂ 0.05 Mann–Whitney test). Tumor size was represented by the value of major diameter (cm).

To evaluate the role of miR-122 and SerpinB3 in vivo, we used the well-characterized chemically induced DEN-HCC rat model, which presents expression profiles representative of human HCC [26]. The results showed an up-regulation of SerpinB3 in HCC tissues with respect to matched surrounding non-tumor samples and a down-regulation of miR-122 in HCC compared to non-tumor samples (Figure 2A,B). In addition, in rat HCC specimens, higher miR-122 levels were associated with lower SerpinB3 expression (Figure 2C), suggesting a possible regulation of SerpinB3 by miR-122 in this animal model. To investigate further the role of serpinB3 in tumor progression, we analyzed the association between SerpinB3 expression and tumor size. In particular, we observed an association between higher SerpinB3 expression and larger tumor size (Figure 2D), supporting the oncogenic function of SerpinB3 in tumor progression. On the contrary, no association between SerpinB3 and caspase-3 or BCl2-modifying factor (BMF) mRNA was detected in rat HCC specimens (Spearman’s rank correlation, *p* = not statistically significant), suggesting that SerpinB3 more likely regulates cell proliferation rather than apoptotic cell death in the DEN-HCC rat model.

**Figure 3:**
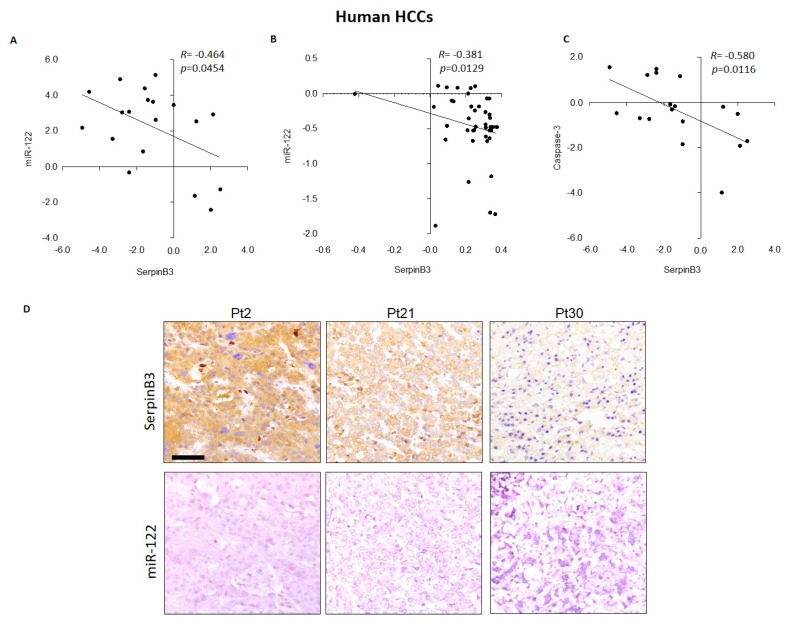
(**A**) Correlation graphs in human HCC tissues between miR-122 and SerpinB3 mRNA and (**B**) protein levels, as detected by qPCR and tissue microarray, respectively. Regarding the qPCR analysis, we considered only HCC samples with a Ct value lower than 35, which resulted in 19 out of 35 tested HCC samples. β-Actin was used as housekeeping gene for SerpinB3 normalization; U6RNA was used as housekeeping gene for miR-122 analysis. (**C**) Correlation graphs in human HCC tissues between SerpinB3 and caspase-3 mRNA levels, as detected by qPCR analysis. (**D**) Representative images of tissue microarray (TMA) in HCC samples from three patients (Pt2, Pt21 and Pt30) with different expression of miR-122 and SerpinB3 in sequential tissue slides. Scale bar 100 µM.

An inverse correlation between miR-122 and SerpinB3 was also found in two HCC patient cohorts, both at mRNA and protein levels, as determined by qPCR and IHC analysis on tissue microarray slides (Figure 3), confirming further the results obtained in preclinical models and strongly suggesting a post-transcriptional regulation of SerpinB3 by miR-122 in HCC. Regarding possible association with clinicopathologic characteristics, we did not find any correlation between SerpinB3 mRNA levels and tumor size, tumor grading, and Alpha-Fetoprotein (AFP) levels. On the contrary, a negative correlation between SerpinB3 and caspase-3 mRNA levels was identified in the first patient cohort (Figure 3C), leading us to hypothesize a role for SerpinB3 in the impairment of apoptosis in the SerpinB3-positive patient subgroup, contributing to their poor prognosis as previously reported by our group [18].

**Figure 4:**
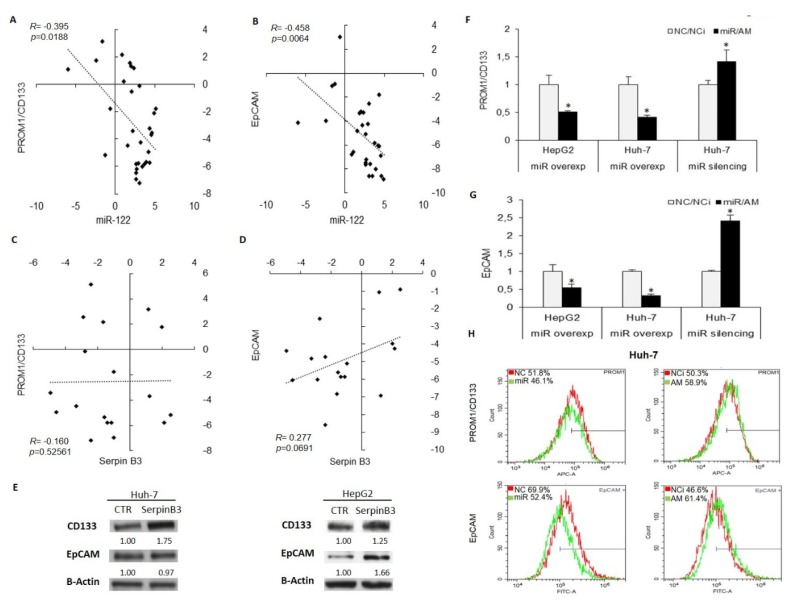
(**A**) Correlation graphs between tissue miR-122 and PROM1/CD133 or (**B**) SerpinB3. (**C**) Correlation graph between EpCAM/CD326 and SerpinB3 or (D) miR-122 levels in the same HCC patient cohort. The X- and Y-axes report 2^−∆∆Ct^ values from qPCR analysis converted in a log_2_ form. U6RNA was used as housekeeping gene for miR-122 normalization, whereas β-Actin was used for gene normalization. (**E**) PROM1/CD133 and EpCAM protein detected by WB analysis in SerpinB3-overexpressing and empty vector-bearing (CTR) HCC cell lines. β-Actin was used as housekeeping gene. (**F**) PROM1/CD133 and (**G**) EpCAM mRNA levels in miR-122-overexpressing (miR) or silenced (AM) HCC cell lines. The Y-axis reports relative values with respect to negative controls. (**H**) Immunophenotype analysis of PROM1/CD133 and EpCAM levels in miR-122 (miR) or anti-miR-122 (AM)-transfected Huh-7 cells. The percentage of positive cells is reported on the top left of the histogram graph. NC: negative control precursor miRNA. NCi: negative control inhibitor miRNA.

Moreover, to investigate further the involvement of miR-122/SerpinB3 axis on hepatocarcinogenesis, we assessed the relationship between miR-122 or SerpinB3 and stemness features in HCC. The expression of stem cell markers PROM1/CD133 and EpCAM/CD326 was analyzed by qPCR in the first patient cohort and in transfected HCC cell lines. MiR-122 inversely correlated with both PROM1/CD133 and EpCAM (Figure 4A,B), while a trend towards a positive correlation was observed between SerpinB3 and EpCAM mRNAs, but not with PROM1/CD133 (Figure 4C,D). In addition, we performed a WB analysis of stem cell markers in SerpinB3-overexpressing Huh-7 and HepG2 cells, chosen because of their well-characterized stemness features [27]. Increased PROM1/CD133 and EpCAM proteins were displayed in SerpinB3-overexpressing HepG2 cells with respect to empty vector cells, whereas SerpinB3-overexpressing Huh-7 cells showed PROM1/CD133 enhanced levels only (Figure 4E), suggesting that SerpinB3 might be, among other miR-122 target genes, in part responsible for miR-122 influence on stem cell characteristics in specific cellular contexts. In this line, decreased PROM1/CD133 and EpCAM levels were displayed in miR-122-overexpressing HepG2 and Huh-7 cells, whereas increased mRNAs levels were detected in miR-122-silenced Huh-7 cells (Figure 4F,G). The immunophenotype of PROM1/CD133 and EpCAM expression in miR-122-overexpressing and silenced Huh-7 cells mirrored mRNA levels (Figure 4H), demonstrating a negative regulation of stem cell features by miR-122 in HCC cells. Taken together, these data demonstrate that miR-122 regulates SerpinB3 and that SerpinB3 contributes, at least in part, to the modulation of stem cell phenotype exerted by the tumor suppressor miR-122 in HCC.

### 3.3. MiR-122 and SerpinB3 Modulate Sorafenib Response in HCC Cell Lines

In order to investigate miR-122 involvement as a possible contributor to sorafenib resistance, HCC-derived cell lines were transfected with pre- or anti-miR-122 and treated with sorafenib for 48 h, and then cell viability and caspase cleavage and activity were evaluated.

Specifically, we chose HepG2 and Hep3B cells for miR-122 overexpression because of their low miR-122 basal levels, while miR-122 silencing was performed in Huh-7 cells due to their high miR-122 basal levels, as previously reported [28].

**Figure 5:**
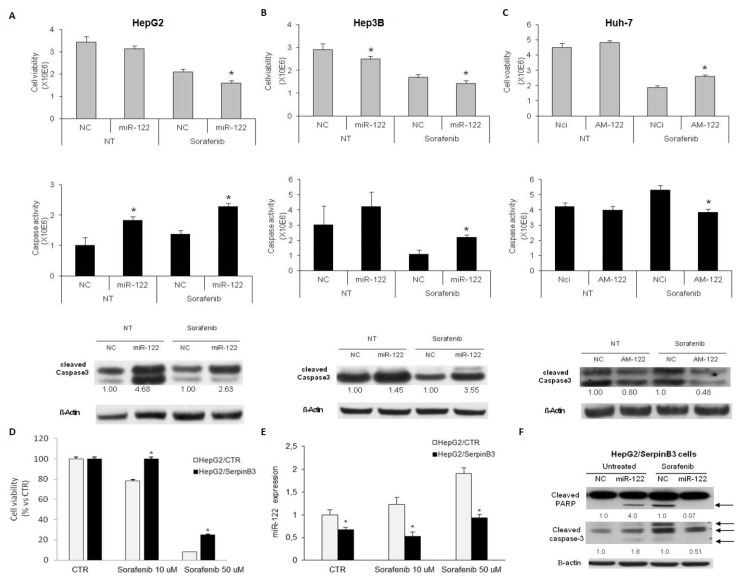
(**A**–**C**) In each panel, from the top to the bottom: cell viability and caspase 3/7 activity assays and western blot analysis of cleaved caspase-3 in HepG2 and Hep3B cells overexpressing miR-122 (**A**,**B**) and in Huh-7 cells transfected with miR-122 inhibitor (AM-122) (**C**) following vehicle (NT) or sorafenib administration. β-Actin was used as housekeeping gene in western blot analysis and numbers represent fold-change values. NC: miRNA precursor negative control; NCi: miRNA inhibitor’s negative control. (**D**) Cell viability assay in SerpinB3-overexpressing HepG2 cells following sorafenib treatment (10 and 50 µM for 48 h). (* *p* ˂ 0.05 Mann–Whitney test). (**E**) MiR-122 quantification in SerpinB3-overexpressing HepG2 cells following sorafenib treatment. (* *p* ˂ 0.05 Mann–Whitney test). (**F**) Western blot analysis of cleaved Poly(ADP-ribose) polymerase 1 (PARP) and cleaved caspase-3 in HepG2 cells overexpressing SerpinB3 (HepG2/SerpinB3) transfected with miR-122 in untreated or sorafenib (10 µM)-treated cells (48 h). β-Actin was used as housekeeping gene in western blot analysis and numbers represent fold-change values. NC: miRNA precursor negative control.

Transient miR-122 overexpression decreased cell viability in sorafenib-treated HepG2 and Hep3B cells and increased apoptotic cell death, as demonstrated by cell viability and caspase 3/7 specific assays and by Western blot analysis of cleaved caspase-3 (Figure 5A,B). Conversely, inhibition of miR-122 caused an increase of cell viability and a decrease of caspase-3/7 activity in Huh-7 cells following sorafenib treatment (Figure 5C). These data confirmed an involvement of miR-122 in sorafenib sensitization of HCC cells, associated with the activation of the caspase signaling.

To evaluate the contribution of SerpinB3 to sorafenib treatment in HCC, stably SerpinB3-overexpressing HepG2 cells were treated with sorafenib and cell viability was evaluated at 48 h. As hypothesized, SerpinB3 overexpression was associated with resistance to sorafenib when compared to empty vector transfected HepG2 cells (Figure 5D). Specifically, a 1.28-fold increase of cell viability was observed in SerpinB3-overexpressing HepG2 cells with respect to empty vector control cells at the 10 µM sorafenib dose, whereas a 4.3-fold increase was observed at the 50 µM sorafenib dose, demonstrating that SerpinB3 overexpression increases sorafenib resistance at different dosages with a stronger effect at the highest dose. Notably, we observed an increase of miR-122 expression following sorafenib treatment in control HepG2 cells only. Conversely, a downregulation of this miRNA occurred in SerpinB3-overexpressing cells in untreated and sorafenib-treated cells with respect to empty vector cells (Figure 5E). Thus, we investigated whether miR-122 downregulation might play a role in sorafenib resistance in HCC cells with SerpinB3 overexpression. As expected, miR-122 transfection in untreated cells increased apoptotic markers, confirming this miRNA as an inducer of apoptosis in HCC cells [28,29] (Figure 5F). On the contrary, miR-122 transfection decreased apoptotic markers in SerpinB3-overexpressing HepG2 cells treated with sorafenib (Figure 5F). These findings do not endorse a further exploitation of combined sorafenib and miR-122 restoration in SerpinB3-positive HCCs.

Data shown are representative results of at least three independent experiments.

## 4. Discussion

MiR-122 is the most abundant miRNA in normal liver and it plays a central role in liver homeostasis, metabolism and differentiation, as well as in HCV replication [30]. Notably, loss of miR-122 has been associated with HCC and its restoration decreases development of HCC in mouse models [12]. While miRNA-122 plays essential roles in the modulation of cancerous phenotype, the mechanisms sustaining its involvement in malignant transformation are still unclear and need to be explored further.

In this study, we identified SerpinB3 as a novel and direct target of miR-122 that might contribute to malignant transformation of hepatocytes. Indeed, our group previously documented the pro-oncogenic role of SerpinB3 [31] and the inverse correlation found in both HCC rat model and human HCC specimens can support the contribution of miR-122/SerpinB3 axis in liver carcinogenesis. Here, we identified an association between SerpinB3 positivity and tumor size in the DEN-HCC rat model as well as a negative correlation between SerpinB3 and caspase-3 mRNA levels in human HCCs, highlighting the contribution of SerpinB3 to the malignant phenotype and tumor progression of HCC. Notably, the high genetic and molecular heterogeneity reported in human HCC might be responsible for the different behavior of SerpinB3 in human and rat HCC specimens. Indeed, while tumors from the DEN-HCC rat model are very homogenous, human HCCs derive from patients with different etiologies, such as HBV/HCV infection and alcohol abuse, which might represent confounding factors influencing both genetic background and tumor growth.

Consistent with previous findings that sorafenib induces apoptotic cell death of HCC cells through the modulation of several pathways and that the identification of new target genes might improve treatment efficacy [32], here we confirmed that miR-122 restoration improves sorafenib sensitivity in HCC cells, reducing their viability and activating the caspase signaling cascade [29]. Since cells overexpressing SerpinB3 are more resistant to sorafenib treatment, we tested whether miR-122 enforced expression might restore sorafenib sensitization in SerpinB3-overexpressing cells. This hypothesis was supported by the evidence that miR-122 overexpression sensitizes HCC cells to chemotherapy [15,28,33], suggesting miR-122 restoration as a possible strategy for HCC and the development of novel technologies for miRNA-targeted delivery as a promising tool for the development of miRNA-based therapeutic options [34]. Our findings outline the role of miR-122 restoration in the induction of apoptosis of HCC cells with different genomic contexts as well as *TP53* mutational status, indicating the high potential of a miR-122-based strategy that might counteract the high heterogeneity of human HCC, which represents a major challenge in terms of treatment effectiveness. However, it should be noted that we could not find in vitro evidence supporting a synergistic effect of miR-122 mimic oligonucleotides with sorafenib in SerpinB3-overexpressing HCC cells, leading us to speculate that their combination might not be effective in SerpinB3-positive HCC patients. Nevertheless, while miR-122 restoration in association with sorafenib did not improve drug sensitization of SerpinB3-overexpressing HCC cells, it is conceivable that other combinations, such as those with *TP53*-interacting chemotherapies, might be more effective in this subgroup of patients. These findings underline the relevance of personalized anticancer treatments to obtain the best therapeutic results.

Hepatic cancer stem cells represent a subpopulation of cells responsible for the tumor initiation and progression and are involved in the metastasis processes and chemoresistance. In line with previous findings, here we confirm the relationship between miR-122/SerpinB3 axis and stemness markers in HCC specimens, as well as the opposite modulation of stem cell features exerted by miR-122 and SerpinB3 in HCC cell lines, suggesting the targeting of SerpinB3 as a possible mechanism contributing to miR-122 active role in reducing HCC stemness. In this context, we previously reported that SerpinB3 is highly expressed in the hepatic stem/progenitor cell compartment of both fetal and adult livers [35]. In addition, an involvement of miR-122 in stem-associated phenotypes was previously reported in HCCs, where it inhibited cancer stem cell (CSC) glycolysis through the direct targeting of PDK4 [36,37,38], suggesting that miR-122 is fundamental for metabolic reprogramming of HCC cells. Moreover, since increased levels of multiple stem cell genes were reported in sorafenib-resistant Huh-7 cells [39], their inhibition by miR-122 mimics might be helpful in reducing sorafenib-acquired resistance when a miRNA replacement therapy is considered.

## 5. Conclusions

In conclusion we identified SerpinB3 as a novel miR-122 target gene that might be, at least in part, responsible for its pivotal role in hepatocarcinogenesis and sorafenib resistance. Moreover, we emphasized the importance of patient-tailored stratification for the identification of combined therapeutic strategies based on miRNA replacement and targeted therapy in advanced HCC patients.

## Supplementary Materials

The following are available online at http://www.mdpi.com/2077-0383/8/2/171/s1, Figure S1. MiR-122 expression in HCC cell lines. Table S1. Primer sequences and PCR conditions.
