# Supplementary material for: MiR-122 Targets SerpinB3 and Is Involved in Sorafenib Resistance in Hepatocellular Carcinoma
Source: J Clin Med. 2019 Feb 1;8(2):171. doi: 10.3390/jcm8020171 (PMC6406326; doi:10.3390/jcm8020171)
Supplement: Supplementary file 1 [file jcm-08-00171-s001.pdf]

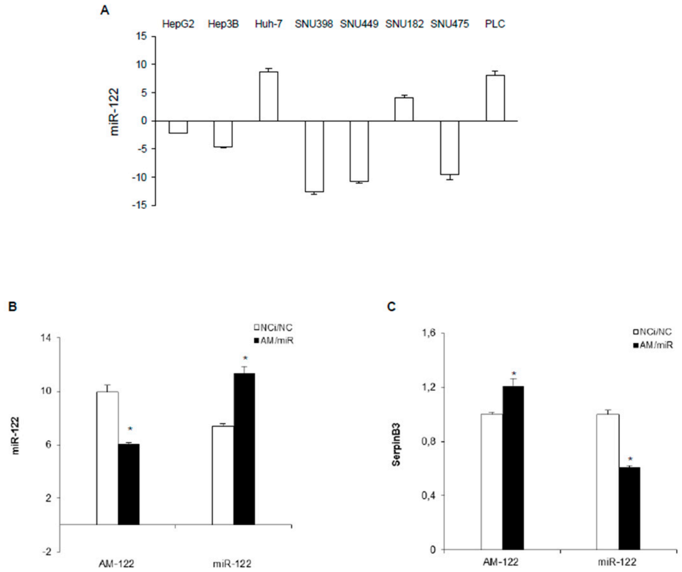

Figure S1. MiR-122 expression in HCC cell lines; (A) qPCR analysis of miR-122 levels in eight HCC cell lines. U6RNA was used as housekeeping gene. Y-axis reports 2-ΔΔCt levels expressed in logarithmic form. (B) qPCR analysis of miR-122 and (C) SerpinB3 levels following miR-122 inhibitor (AM-122) or miR-122 mimic transfection in Huh-7 cells stably overexpressing SerpinB3 (Huh-7 SB3), with respect to controls. U6RNA and ß-Actin were used as housekeeping genes. Y-axis reports $2^{-\Delta\Delta Ct}$ miR-122 levels expressed in logarithmic form and relative SerpinB3 expression levels. NCi: miRNA inhibitors negative control; NC: miRNA precursor negative control. (* $p < 0.05$).

Table 1. Primer sequences and PCR conditions.

Gene name	Species	Primer Sequences	PCR product (bp)	Anneling T (°C)	Cycles
SerpinB3	Homo sapiens	Fw 5'-AACTCCTGGGTGGAAAGTCAA-3'<br>Rv 5'-ACCAATGTGGTATTGCTGCCAA-3'	83	60	45
EpCam	Homo sapiens	Fw 5'-CAATGCAGGGTCTAAAAGCTG-3'<br>Rv 5'-CACCCATCTCCTTTATCTCAGC-3'	140	61.8	45
CD133	Homo sapiens	Fw 5'-GTGGATGCAGAACTTGACAA-3'<br>Rv 5'-ACCCTTTTGATACCTGCTACG-3'	117	61.8	45
SerpinB3	Rattus Norvegicus	Fw 5'-GAGGTACAGATCGCCAGAAAG-3'<br>Rv 5'-TCCCTGTTTTCTCGTCTGTTC-3'	149	61.8	45
GAPDH	Homo sapiens	Fw 5'-TGGTATCGTGGAAGGACTCATGAC-3'<br>Rv 5'-ATGCCAGTGAGCTTCCCGTTCAGC-3'	189	60	45
ß-ACTIN	Rattus Norvegicus	Fw 5'-CACTTTCTACAATGAGCTGCG-3'<br>Rv 5'-CTGGATGGCTACGTACATGG-3'	147	60	45
SerpinB3 3'UTR	Homo sapiens	Fw 5'-**AATCTAGA**AGAGAAACTCACTGC-3'<br>Rv 5'-**ATCTAGA**GAAAGAGAGAAAGGCATGC-3'	587	59.5	40
